# Safety and efficacy of sorafenib in patients with advanced hepatocellular carcinoma and Child-Pugh A or B cirrhosis

**DOI:** 10.3892/ol.2015.2960

**Published:** 2015-02-12

**Authors:** ALESSANDRO FEDERICO, MICHELE ORDITURA, GAETANO COTTICELLI, ILARIO DE SIO, MARCO ROMANO, ANTONIETTA GERARDA GRAVINA, MARCELLO DALLIO, ALESSIO FABOZZI, FORTUNATO CIARDIELLO, CARMELA LOGUERCIO, FERDINANDO DE VITA

**Affiliations:** 1 Division of Hepatogastroenterology, Department of Clinical and Experimental Medicine, Second University of Naples, Naples 80131, Italy; 2 Division of Oncology, Department of Clinical and Experimental Medicine, Second University of Naples, Naples 80131, Italy

**Keywords:** sorafenib, hepatocellular carcinoma, systemic treatment, targeted therapy

## Abstract

Sorafenib confers a survival benefit for patients with advanced hepatocellular carcinoma (HCC) and Child-Pugh (CP) A liver cirrhosis. At present, limited data exists with regard to the safety and efficacy of sorafenib in treating CP-B HCC patients. The present study describes the use of sorafenib in patients with HCC and CP-A or -B cirrhosis. Clinical data was obtained from patients with HCC who were treated with sorafenib at the Department of Clinical and Experimental Medicine, Second University of Naples (Naples, Italy) and were analyzed retrospectively in terms of tumor response, tolerance and survival. The treatment outcomes were analyzed according to the respective CP status. The adverse events (AEs) were graded using the Common Terminology Criteria for Adverse Events, version 3.0, and the tumor response was assessed according to the Response Evaluation Criteria in Solid Tumors, version 1.2. In total, 26 patients received sorafenib at 400 mg twice daily. The median age was 69 years (range, 58–81 years) and the ratio of males to females was 18:8. Overall, 15 patients were infected with the hepatitis C virus (HCV), eight with HBV and three were co-infected with HCV/HBV. In total, 20 (77%) patients presented with an underlying CP-A (CP-A5 and CP-A6) cirrhosis and six (23%) with CP-B (CP-B7). Previous treatments included surgery (n=4), transarterial chemoembolization (n=5) and percutaneous ethanol injection or radiofrequency interstitial thermal ablation (n=12). A partial response was observed in three patients (12%), a stable disease lasting at least 12 weeks in 13 patients (50%) and a progression of disease in 10 patients (38%). The median overall survival (OS) time was 7.4 months [95% confidence interval (CI), 3.2–11.6) and the median progression-free survival (PFS) time was 3.7 months (95% CI, 1.9–5.5). The median OS and PFS times differed between patients with CP-A and CP-B, with a trend (P=0.06) toward a worse outcome in those with CP-B, although this was not statistically significant. The CP-A and CP-B groups experienced a similar incidence in the majority of AEs. A reduction in dose was required in 59% of the patients. The CP-A5, CP-A6 and CP-B7 patients tolerated sorafenib similarly, and derived comparable clinical and survival benefits.

## Introduction

Hepatocellular carcinoma (HCC) is the third major cause of cancer-related mortality, and the fifth most common type of cancer worldwide. In total, ≤85% of HCC cases occur in patients with underlying liver cirrhosis. The primary causes of chronic liver insufficiency in Europe are those of viral etiology, namely hepatitis B virus (HBV) and HCV, and alcoholic and non-alcoholic steatohepatitis (1). At present, the overall survival (OS) rate for patients with HCC remains poor, with 26% of patients with early-stage disease and 2% of those with advanced-stage disease surviving for more than five years (2). The tumor stage and level of liver function are extremely important factors to be considered for the therapeutic strategy and final outcomes of HCC (3). In certain Western countries, ~10% of patients with HCC are diagnosed at an advanced disease stage, and just 30% are eligible for potentially curative therapies. Furthermore, a marked percentage of patients treated for early HCC progress to an advanced stage of the disease (4,5). Sorafenib (Nexavar^©^; Bayer Health Care, Leverkusen, Germany) is a multi-kinase inhibitor that blocks cell proliferation and angiogenesis by inhibiting, among others, the Raf-1 and B-Raf serine/threonine protein kinases and the tyrosine kinase activity of the vascular endothelial growth factor receptor (VEGFR) (types 1–3) and the β-type platelet-derived growth factor receptor (PDGFRβ), all of which are involved in key intracellular signaling pathways during hepatic carcinogenesis (6–8). Previously, two randomized phase III studies demonstrated a significant advantage in terms of progression-free survival (PFS) and OS following treatment with sorafenib versus a placebo (9,10), a result which was later confirmed in a meta-analysis (11). The patients involved in the aforementioned phase III studies were diagnosed with Child-Pugh (CP)-A liver disease, while patients with a CP-B score and poor liver function (total bilirubin, >3 mg/dl; international normalized ratio, >2.3 and albumin, <2.8 g/dl) were systematically excluded. However, this does not reflect the reality of clinical practice, and to date, limited data regarding the efficacy and tolerability of sorafenib in patients with major liver dysfunction (CP-B or -C) has been published. Due to the poor prognoses of patients with CP-C, including a short life expectancy and a high risk of deterioration, treatment with sorafenib is not currently recommended for this subgroup (12). For patients with CP-B, an expert consensus concluded that treatment should be individualized, with particular attention paid to the liver function status (13). While CP-A and C are clearly defined on this basis as compensated or decompensated stages, respectively, CP-B includes compensated and decompensated patients, and therefore constitutes a heterogeneous population (13). Results obtained from a previous phase II trial demonstrated that the pharmacokinetic outcomes and side-effects were comparable between individuals with CP-A and -B liver disease, and that a dose reduction was not recommended (14). By contrast, a phase I trial, which analyzed the efficacy of sorafenib in patients with solid tumors and impaired liver or renal function (15), determined that treatment should be administered at a lower dose initially in case of elevated bilirubin levels and in order to avoid dose-limiting toxicity. A pioneering clinical trial by Abou-Alfa (16), which examined the effects of sorafenib in patients with HCC, identified differences between the liver function of patients with CP-A and -B. Those with CP-B presented more often with bilirubin elevation, ascites, encephalopathy and a shorter OS time (41 weeks vs. 14 weeks). Additional case series and retrospective studies have also been published. Zugazagoitia *et al* (3) described a group of CP-A and -B patients who were treated with sorafenib, with the aim of establishing the efficacy and safety of the drug, and to compare the results of the patients with CP-A or -B. In this study, it was demonstrated that the OS time was significantly longer for patients with CP-A than those with CP-B liver disease (8.7 vs. 4.7 months, respectively). Furthermore, grade (G)4 liver-related events primarily occurred at the time of sorafenib initiation in CP-B patients with decompensated cirrhosis. In a previous retrospective analysis, Chiu *et al* (17) examined the use of sorafenib for the treatment of advanced HCC with underlying CP-B liver cirrhosis. The results revealed that patients with either CP-A or -B tolerated sorafenib similarly, and derived similar clinical and PFS benefits. However, those patients with CP-B liver disease demonstrated an increased susceptibility to developing cirrhotic complications.

Therefore, a requirement exists to establish the efficacy and safety of sorafenib in CP-B patients, and to identify homogeneous subgroups who could benefit from this treatment. The primary aim of the present study was to evaluate the efficacy and safety of sorafenib in CP-A and -B patients via the analysis of daily clinical conditions and the comparison of results in terms of efficacy and toxicity. The second aim was to compare these results between compensated and decompensated CP-B patients, and to identify whether this variable could aid in defining a subgroup of CP-B patients who could benefit from treatment with sorafenib.

## Materials and methods

Clinical, biological and radiological data from all patients consecutively treated with sorafenib for advanced HCC at the Divisions of Hepatogastroenterology and Oncology at the Second University of Naples (Naples, Italy) between 2010 and 2012 were prospectively collected. Informed consent was obtained from each patient prior to treatment with sorafenib. Due to a poor prognosis and advanced liver dysfunction, patients with CP-B8, CP-B9 and -C liver disease were excluded from sorafenib therapy. A diagnosis of HCC was made using the criteria of the European Association for the Study of the Liver (5) and the American Association for the Study of Liver Disease (4), or results from the pathological analysis. The tumor stage was established according to the Barcelona Clinic Liver Cancer staging system (18). Patients who had received prior loco-regional treatment, including transcatheterarterial chemoembolization (TACE), radiofrequency ablation (RFA) and percutaneous alcohol injection (PEI), or hormonal therapy, were accepted in the present study. A platelet count of >60×10^9^/l, a neutrophil count of >10×10^5^/l, the presence of adequate left ventricular function and the absence of any severe thromboembolic or bleeding events in the previous six months was required in order for the patient to be eligible for sorafenib therapy. Sorafenib was administered by continuous oral doses of 400 mg twice daily. Each cycle was defined as four consecutive weeks of treatment. Contrast-enhanced computed tomography (CT) was performed every three cycles, and the tumor response was assessed according to the Response Evaluation Criteria in Solid Tumors, version 1.2 (19). The primary predicted adverse events (AEs) of hand-foot syndrome, diarrhea, fatigue and the presence of a rash, were prospectively registered at baseline and monitored every month thereafter according to the National Cancer Institute Common Terminology Criteria for Adverse Events, version 3.0 (19). Additional toxicities were also recorded, but only if they exhibited a severity greater than G2. Those patients who were not able to tolerate the full-dose treatment, or those who developed G3 or higher toxicities, were permitted treatment interruption or a dose reduction. In total, two dose reductions were authorized, first to 400 mg daily, and then to 200 mg daily. The treatment was continued until the incidence of disease-progression, AEs, complications or mortality. The patients were assessed every four weeks by clinical examination, blood tests and ultrasonography, until mortality or the last follow-up date. A total of 24 months of follow-up was planned. Patients were evaluated by helicoidal CT scanning or magnetic resonance imaging four weeks prior to the start of treatment and every three months thereafter.

Events associated with liver failure or liver decompensation, such as the development or worsening of hyperbilirubinemia, ascites or encephalopathy, were analyzed separately. From the patients with CP-B liver disease, two subgroups were established based upon whether the cirrhosis was compensated or decompensated at the start of sorafenib treatment, which was determined by the presence of ascites, clinical hepatic encephalopathy or jaundice (total bilirubin serological value, >3 mg/dl).

## Results

**Figure 1:**
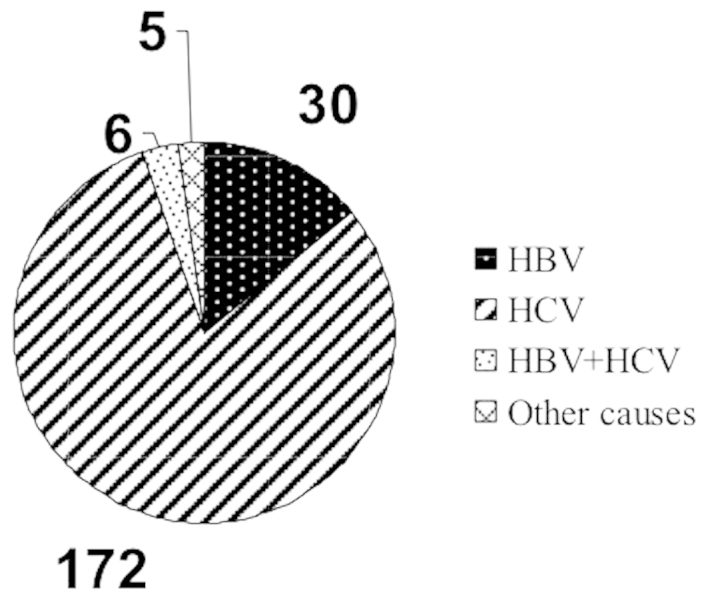
Number of patients with varying etiologies of liver injury. HBV, hepatitis B virus; HBC, hepatitis C virus.

**Figure 2:**
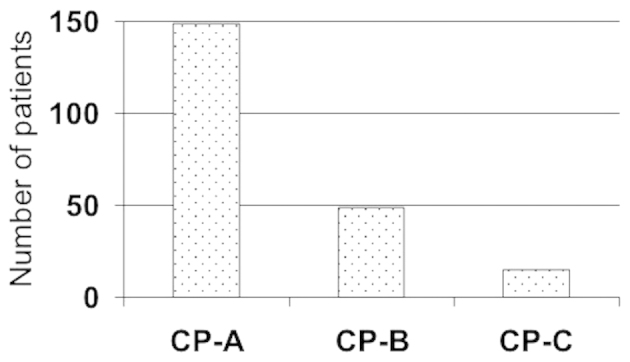
Number of patients with different Child-Pugh (CP) liver disease scores.

A total of 213 patients with primary HCC were evaluated at the Department of Clinical and Experimental Medicine, Second University of Naples between 2010 and 2012. The etiology of liver injury was investigated and is shown in Fig. 1. Overall, infection with hepatitis C virus (HCV) was the primary cause of liver disease (81%), followed by HBV (14%), HBV-HCV co-infection (3%) and other causes, such as those resulting from non-alcoholic fatty liver disease/non-alcoholic steatohepatitis, hemochromatosis or those of a cryptogenic origin (2%). In total, 149 out of 213 patients presented with CP-A liver disease, 49 with CP-B and 15 with CP-C (Fig. 2). The median value of the Model for End-Stage Liver Disease score was eight (5). A specific treatment approach was proposed for all patients with HCC (4,5): 70 patients underwent RFA, 64 received PEI, 12 received TACE and 11 underwent surgical resection; 30 patients were untreated according to International Guidelines; and 26 patients were eligible for sorafenib treatment and began therapy at 400 mg twice daily. Treatment was continued until unacceptable toxicity, mortality, patient refusal or loss to follow-up. The patient characteristics are shown in Table I.

The median treatment duration was 255 days (range, 15 to 730 days). The average median time for treatment was 386.3 days. Patients with CP-A and -B liver disease had a median treatment time of 178 days and 621 days, respectively.

A dose reduction was required in 59% of the patients due to AEs. In total, five patients stopped sorafenib treatment due to unacceptable toxicity or adverse reactions, while six patients stopped due to disease progression. One patient was lost to follow-up.

A partial response (PR) was observed in three of the patients (12%), stable disease (SD) lasting at least 12 weeks was observed in 13 patients (50%) and progression of disease (PD) was observed in 10 patients (38%). The median OS and PFS times were 7.4 (95% CI, 3.2–11.6) and 3.7 (95% CI, 1.9–5.5) months, respectively. The median OS and PFS times differed between patients with CP-A5–6 and -B7 liver disease patients, with a trend (P=0.06) toward a worse outcome in CP-B patients, although this was not statistically significant. Patients without extra-hepatic spread, particularly those without lung metastasis, were more likely to benefit from sorafenib treatment. In total, ~35% of the patients demonstrated G1–2 toxicity, characterized primarily by diarrhea, malaise and skin reactions. Only two patients (9%) experienced a G3–4 toxicity, characterized by diarrhea and hematological disorder. The most common G3 toxicities were hand-foot-skin reactions (23%), malaise (15%), diarrhea (7%) and mucositis (4%). In addition, 12 patients (46%) experienced transient liver function derangement. Overall, the two groups of patients (CP-A and CP-B7) demonstrated similar incidences of AEs. No differences were identified between the therapeutic benefits and toxicity, following sorafenib treatment, among patients with and without underlying portal vein thrombosis.

## Discussion

Despite the recent improvements made in the loco-regional treatment and management of advanced HCC, its prognosis remains poor. In the last decade, a number of drugs have been tested in clinical and pre-clinical studies, but only a few compounds have conferred a significant improvement in patient survival for advanced disease (8). Since 2008, sorafenib has represented the only therapeutic agent able to positively impact upon the survival of patients with advanced HCC with preserved liver function. Sorafenib is a small, multi-target inhibitor, which simultaneously blocks the RAF-MEK-ERK pathway to prevent tumor growth, and inhibits the action of VEGFR1, 2 and 3 and PDGFR-β to suppress neoangiogenesis (10).

In the present study, 26 patients with advanced HCC were treated with sorafenib at the standard dose of 400 mg twice daily for a median time of 255 days, and up to progression of disease or unacceptable toxicity. Subsequent to a median follow-up period of 262.5 days, a median OS time of 7.4 months, with a slight advantage for the patients without extra liver spread of the disease, and a median PFS time of 3.7 months was observed. Furthermore, in the treated population it was revealed that 11 and 50% of patients demonstrated a PR and SD for at least 12 weeks, respectively. These results are notable, particularly when compared with the results reported in larger trials. A 2009 double-blind phase III trial by Cheng *et al* (10) randomized 226 patients with advanced HCC to receive either sorafenib at a dose of 400 mg twice daily or a placebo. In the experimental arm, a median OS time of 6.5 months (95% CI, 5.56–7.56) and a median time to progression of 2.8 months (95% CI, 2.63–3.58) was documented. Similarly, in the larger double blind phase III Study of Heart and Renal Protection trial (SHARP), Llovet *et al* (9) randomized 602 patients with advanced HCC to receive either sorafenib at a dose of 400 mg twice daily or a placebo. In the experimental arm, a median OS time of 10.7 months was achieved.

The benefits conferred by sorafenib, in terms of OS and PFS time, have been confirmed in all subgroups of patients. In particular, notable findings have been observed in CP-B patients. Firstly, the results from the present study did not support a worse safety profile in this sub-group. Furthermore, no statistically significant differences were identified between the patients with CP-A or -B liver cirrhosis. Therefore, these results support the use of sorafenib, at least in patients with CP-B7 (the CP-B classification found in the present study), with similar results in terms of efficacy and safety compared with CP-A patients.

The available literature regarding HCC, CP score and the use of sorafenib is discordant. Previously, it has been demonstrated that in certain patients with advanced HCC, the CP-B score is predictive of a poorer outcome (20). This finding can be attributed to high liver dysfunction and to the most compromised condition of the patients, rather than the effect of the drug. In addition, two retrospective analyses and a case-control study revealed that advanced HCC CP-B patients treated with sorafenib exhibited a poorer outcome than those with CP-A liver disease (14,21,22). Sorafenib appears to be effective for those CP-B patients with less compromised liver functionality, with a similar toxicity profile observed in compensated and decompensated subgroups. These data confirm the viability of sorafenib in this population, even if they require future confirmation (21–24).

With regard to the safety analysis in the treated population, it was revealed that 35% of patients experienced G1–G2 toxicities, with the most common symptoms including diarrhea, asthenia and skin reactions. In total, 9% of patients experienced G3–G4 effects, namely hand-foot skin reactions, asthenia, diarrhea and mucositis. In addition, the results from the present study appear to be in line with data reported by previous studies, although the number of cases in the present study is limited. In fact, in an Asiatic trial (10), the most common G3–G4 AEs were hand-foot skin reactions (10.7%) and diarrhea (6%). By contrast, in the SHARP trial (9), hand-foot skin reactions and diarrhea were only observed in 2.7% of cases. Despite previous studies having identified an increase in hemorrhagic risk and cardiac events associated with the use of sorafenib (25), similar toxicities were not observed in the present study.

In the present study, the incidence of AEs lead to the suspension of drug administration in 9.2% of the patients, and a dose reduction in 59%. These changes may have affected the treatment efficacy of sorafenib and induced a sub-optimal pharmacokinetic outcome. The results presented from a recent global investigation of therapeutic decisions in hepatocellular carcinoma and of its treatment with sorafenib trial (26), even if preliminary, confirmed that a full dose of sorafenib could be predictive of an improved patient response. Therefore, if possible, treatment should be continued for as long as possible, and only stopped or the dose reduced in the case of serious AEs (24).

## Figures and Tables

**Table I:** Characteristics of the 26 patients eligible for sorafenib treatment.

Characteristics	Value
Gender, n (%)	
Male	18 (69)
Female	8 (31)
Median age, years (range)	69 (58–81)
Underlying liver disease, n (%)	
HCV	15 (58)
HBV	8 (31)
HCV and HBV	3 (11)
Child-Pugh classification, n (%)	
A5–6	20 (77)
B7	6 (23)
Previous treatments for HCC, n (%)	
No treatment	5 (19)
Resection	4 (16)
TACE	5 (19)
PEI/RFA	12 (46)

HCV, hepatitis C virus; HBV, hepatitis B virus; HCC, hepatocellular carcinoma; TACE, transarterial chemoembolization; PEI, percutaneous ethanol injection; RFA, radiofrequency interstitial thermal ablation.

## References

[1] El-Serag HB, Rudolph KL (2007). Hepatocellular carcinoma: epidemiology and molecular carcinogenesis. Gastroenterology.

[2] American Cancer Society (2010). Cancer facts and figures 2010.

[3] Zugazagoitia J, Manzano A, Sastre J, Ladero JM, Puente J, Dìaz-Rubio E (2013). Sorafenib for non-selected patient population with advanced hepatocellular carcinoma: efficacy and safety data according to liver function. Clin Transl Oncol.

[4] Bruix J, Sherman M, Practice Guidelines Committee, American Association for the Study of Liver Diseases (2005). Management of hepatocellular carcinoma. Hepatology.

[5] Bruix J, Sherman M, Llovet JM, EASL Panel of Experts on HCC (2001). Clinical management of hepatocellular carcinoma. Conclusions of the Barcelona-2000 EASL Conference. European Association for the Study of the Liver. J Hepatol.

[6] Wilhelm SM, Carter C, Tang L (2004). BAY 43–9006 exhibits broad spectrum oral antitumor activity and targets the RAF/MEK/ERK pathway and receptor tyrosine-kinases involved in tumor progression and angiogenesis. Cancer Res.

[7] Villanueva A, Newell P, Chiang DY, Friedman SL, Llovet JM (2007). Genomics and signaling pathways in hepatocellular carcinoma. Semin Liver Dis.

[8] Kaneko S, Furuse J, Kudo M (2012). Guideline on the use of new anticancer drugs for the treatment of hepatocellular carcinoma 2010 update. Hepatology Res.

[9] Llovet JM, Ricci S, Mazzaferro V, SHARP Investigators Study Group (2008). Sorafenib in advanced hepatocellular carcinoma. N Engl J Med.

[10] Cheng AL, Kang YK, Chen Z (2009). Efficacy and safety of sorafenib in patients in the Asia-Pacific region with advanced hepatocellular carcinoma: a phase III randomized, double-blind, placebo-controlled trial. Lancet Oncol.

[11] Kim BK, Kim SU, Park JY (2012). Applicability of BCLC stage for prognostic stratification in comparison with other staging systems: single centre experience from long-term clinical outcomes of 1717 treatment-naïve patients with hepatocellular carcinoma. Liver Int.

[12] Wörns MA, Weinmann A, Pfingst K (2009). Safety and efficacy of sorafenib in patients with advanced hepatocellular carcinoma in consideration of concomitant stage of liver cirrhosis. J Clin Gastroenterol.

[13] Peck-Radosavljevic M, Greten TF, Lammer J, Rosmorduc O, Sangro B, Santoro A, Bolondi L (2010). Consensus on the current use of sorafenib for the treatment of hepatocellular carcinoma. Eur J Gastroenterol Hepatol.

[14] Abou-Alfa GK, Amadori D, Santoro A (2011). Safety and efficacy of sorafenib in patients with hepatocellular carcinoma (HCC) and Child-Pugh A versus B cirrhosis. Gastrointest Cancer Res.

[15] Miller AA, Murry DJ, Owzar K (2009). Phase I and pharmacokinetic study of sorafenib in patients with hepatic or renal dysfunction: CALGB 60301. J Clin Oncol.

[16] Abou-Alfa GK (2009). Commentary: Sorafenib - the end of a long journey in search of systemic therapy for hepatocellular carci- noma, or the beginning?. Oncologist.

[17] Chiu J, Tang JF, Yao TJ (2012). The use of single-agent sorafenib in the treatment of advanced hepatocellular carcinoma patients with underlying Child-Pugh B liver cirrhosis: a retrospective analysis of efficacy, safety, and survival benefits. Cancer.

[18] Llovet JM, Brú C, Bruix J (1999). Prognosis of hepatocellular carcinoma: the BCLC staging classification. Semin Liver Dis.

[19] Therasse P, Arbuck SG, Eisenhauer EA (2000). New guidelines to evaluate the response to treatment in solid tumors. European Organization for Research and Treatment of Cancer, National Cancer Institute of the United States, National Cancer Institute of Canada. J Natl Cancer Inst.

[20] Tandon P, Garcia-Tsao G (2009). Prognostic indicators in hepatocellular carcinoma: a systematic review of 72 studies. Liver Int.

[21] Kim JE, Ryoo BY, Ryu MH (2011). Sorafenib for hepatocellular carcinoma according to Child-Pugh class of liver function. Cancer Chemother Pharmacol.

[22] Hollebecque A, Cattan S, Romano O (2011). Safety and efficacy of sorafenib in hepatocellular carcinoma: the impact of the Child-Pugh score. Aliment Pharmacol Ther.

[23] Pinter M, Sieghart W, Graziadei I (2009). Sorafenib in unresectable hepatocellular carcinoma from mild to advanced stage liver cirrhosis. Oncologist.

[24] Iavarone M, Cabibbo G, Piscaglia F, SOFIA (SOraFenib Italian Assessment) study group (2011). Field-practice study of sorafenib therapy for hepatocellular carcinoma: a prospective multicenter study in Italy. Hepatology.

[25] Je Y, Schutz FA, Choueiri TK (2009). Risk of bleeding with vascular endothelial growth factor receptor tyrosine-kinase inhibitors sunitinib and sorafenib: a systematic review and meta-analysis of clinical trials. Lancet Oncol.

[26] Lencioni R, Kudo M, Ye SL (2014). GIDEON (Global Investigation of therapeutic DEcisions in hepatocellular carcinoma and Of its treatment with sorafeNib): second interim analysis. Int J Clin Pract.

